# Community members’ perceptions of community health workers in Melusi, Tshwane, South Africa

**DOI:** 10.4102/phcfm.v16i1.4573

**Published:** 2024-09-19

**Authors:** Rebaone K. Madzivhandila, Sanele Ngcobo

**Affiliations:** 1 School of Health Systems and Public Health, Faculty of Health Science, University of Pretoria, South Africa; 2 Department of Family Medicine, Faculty of Health Science, University of Pretoria, Pretoria, South Africa

**Keywords:** community health workers, health promotion, informal settlement, community perception, barriers, healthcare accessibility

## Abstract

**Background:**

Community health workers (CHWs) play an important role in health promotion through health campaigns and home visits, and provide linkage to care and support patient management. In the informal settlements, CHWs identify health challenges and help residents to access healthcare facilities.

**Aim:**

This study aimed to explore the perception held by the community members regarding CHWs’ contribution to health promotion in Melusi informal settlements within the Tshwane district.

**Setting:**

The study was conducted in Melusi informal settlement situated in Pretoria west in South Africa.

**Methods:**

Descriptive exploratory qualitative research methods were used, with open and closed-ended questions during individual interview sessions. Participants were purposefully selected in collaborating with the community leaders and using CHWs’ data to identify individuals with direct interaction with CHWs.

**Results:**

Participants praised CHWs’ communication skills, engagement strategies, and ability to provide valuable health services. Community members expected CHWs to provide healthcare services, educational support, advocacy for health issues and guidance on well-being. Barriers such as community resistance, a lack of privacy and difficulties in reaching households were noticed. However, CHWs were commended for their impact on well-being, offering health services, emergency support, food assistance and improved healthcare accessibility.

**Conclusion:**

The study indicates that community members have positive perceptions of CHWs. This highlights the significant role of CHWs in promoting health and well-being within the community.

**Contribution:**

This study provides insights into community perceptions of CHWs in informal settlements, emphasising their impact on community well-being. It provides a basis for their effectiveness and support in delivering health services in the informal settlement.

## Introduction

The City of Tshwane has a number of informal settlements where communities are faced with healthcare challenges. In these settlements, residents usually experience health-related issues, limited access to formal healthcare and a lack of service delivery. The role of community health workers (CHWs) is vital in health promotion given the need for community-driven healthcare solutions. The CHWs are an integral part of the Community Orientated Primary Care (COPC) approach.^1^ They must establish relationships with both household members and community leaders to build the link between community health and community members.^1^

The CHWs are trained and equipped with various skills and taught how to implement the training practically.^2^ They are instrumental in ensuring the healthcare of individuals in the community.^2^

They employ various methods, such as health promotion, counselling and education to individuals and households within the community.^2^

Low- and middle-income countries face several challenges in providing basic healthcare to their populations, primarily because of financial, infrastructural and human resource constraints.^3^ The CHWs usually provide affordable basic healthcare to the people in their communities.^3^ They are in a good position to connect the community with the healthcare system and make sure people get the care they need consistently.^3^ The CHW programmes vary in design, ranging from large government initiatives to smaller community-led programmes.^3^ These programmes have made it easier for people in remote areas to access healthcare services.^3^

Community perceptions play an important role in understanding the effectiveness and acceptance of CHWs and their role in health promotion within the community.^3^ Community perceptions of CHWs, whether positive or negative, can provide valuable feedback for decision-making in healthcare policies and resource allocation.^3^

This study explored the perceptions held by the community members residing in the Melusi informal settlement in the City of Tshwane district.

## Research methods and design

### Study design

A descriptive exploratory qualitative study design was used to uncover the deeper meanings and essence of residents’ perceptions regarding CHW’s roles in health promotion within informal settlements, providing valuable insights for improving community-oriented healthcare interventions. The study used both open and closed-ended questions when conducting in-depth interviews. This study examined the lived experiences and perceptions of residents residing in Melusi informal settlements regarding the role of CHWs in health promotion.

### Setting

The study took place in Melusi informal settlement in Pretoria West within the City of Tshwane District, South Africa. Melusi is a growing informal settlement located within Greater Tshwane Metropolitan Municipality in the Gauteng province.^4^ The area is divided into three sections, namely Melusi 1, 2, and 3, commonly known as Gomora, Malaribeng and Maluleka, respectively, with approximately 8000 households.

The primary healthcare services for this community are rendered at Daspoort Poli-Clinic and Hercules Clinic while Pretoria West Hospital and Kalafong provides the secondary healthcare services.

The Melusi community is served by 22 CHWs who conduct household visits and provide assistance at the local clinic. Each CHW is assigned to approximately 250 households, where they engage in a wide range of health promotion activities and preventive measures. Their responsibilities include identifying individuals with poor treatment compliance and providing education to enhance adherence to medical regimens. Beyond this, CHWs play a crucial role in offering basic health education, promoting healthy lifestyles and advocating for public health initiatives within the community. They assist in the management of chronic diseases by monitoring patients’ health status and ensuring continuity of care. The CHWs also provide psychosocial support to patients and their families, helping them navigate the healthcare system and access necessary services. They are instrumental in mobilising community resources and fostering community participation in health-related activities. Their efforts include organising health campaigns, conducting workshops and disseminating information on disease prevention and health maintenance.

The community operates a satellite clinic led by a clinical associate and a nurse, offering acute management and screening for both non-communicable and communicable diseases. This clinic functions twice a week, with two CHWs rotating to assist in various tasks, such as taking vital signs, weighing infants, conducting human immunodeficiency virus (HIV) tests and linking patients to the nearest primary healthcare facility for chronic disease services.

### Study population and sampling strategy

The study participants were residents of the Melusi informal settlement. These individuals represented the target group whose perceptions regarding the role of CHWs in health promotion were investigated. The recruitment process for this study followed a carefully planned approach to identify and select participants who align with the inclusion criteria. The criteria for inclusion encompassed individuals aged 18 years and above residing in Melusi, who had direct exposure to CHWs and health promotion activities. Candidates were excluded if they lacked direct interaction with CHWs or if they were visiting but not residing within the Melusi area.

The sample size for this study was determined through a process guided by the study’s qualitative design, specifically aiming to achieve data saturation where no new information or themes emerge from additional data collection. Following guidelines in the qualitative research literature by Guest et al. and Saunders et al., which suggest that a minimum of 12 to 20 participants is often sufficient to achieve data saturation, we focussed on including a diverse group of participants from the Melusi informal settlement.^5,6^ Throughout the study, we closely monitored data saturation and data collection continued until no new insights emerged from the interviews.

Potential participants were identified through purposeful sampling, involving collaborating with community leaders, local organisations and data collected by CHWs using the COPC approach. Households listed on the CHWs’ records as receiving care were visited. During these visits, the head of each household was informed about the study and invited to nominate an adult participant who had experience with CHWs. This approach ensured that participants selected for the study were knowledgeable about the role of CHWs and had relevant experiences to contribute.

After recruitment, the researcher initiated contact with the participants and then clarified the study’s objectives. A clear overview of the research aims and the importance of their perspectives was provided during the initial communication. Individuals expressing interest in participating were provided with a detailed informed consent form. Participants had the opportunity to ask questions and only proceed if they voluntarily provided written consent.

### Data collection

A semi-structured interview was used for data collection, aiming to get detailed information on the community’s perception of CHWs’ role in health promotion. The interview guide was developed by the researcher aligning with the study’s aim and focussing on how community members perceive CHWs’ impact on the health outcome in the informal settlements in the Tshwane district (Supplementary File 1). The researcher used the interview guide during the data collection to ensure consistency throughout the data collection process. Interviews were conducted either at the participant’s household or at the Melusi Clinic for those unable to host at home. The interviews took place in person and were conducted in the participants’ home language to facilitate open and meaningful communication. Participants were encouraged to freely express their views and opinions in their preferred language. The process began with providing participants comprehensive information about the study, allowing them to ask questions to ensure clarity. Once participants fully understood the study, they were presented with a consent form to sign if they chose to participate. The primary question posed to participants was, ‘Do you believe that CHWs play a significant role in addressing these health challenges? Why or why not?’ Probing questions were used to delve deeper into responses (Supplementary File 1).

Participants were asked a combination of open-ended and close-ended questions to explore their experiences thoroughly. All information collected during the interviews was recorded and field notes were documented both during and after each interview to ensure the accuracy and integrity of the data.

### Data analysis

The transcription process followed guidelines for qualitative research methodologies by Guest et al., ensuring accuracy, context preservation and consistency throughout.^7^ Bilingual transcribers, a research assistant at the School of Public Health with experience in transcribing, proficient in both the participants’ home languages and the study language, initially transcribed audio recordings in the participants’ native languages to capture their exact expressions and wording. These transcripts were then translated into the study language to maintain context, preserve meaning, support analysis and ensure a comprehensive understanding of participants’ views and opinions. The researcher meticulously reviewed transcripts and repeatedly listened to recordings to verify their accuracy throughout the transcription and translation process.

To analyse the data, the first author, RKM, used ATLAS.ti Scientific Software Development GmbH, Berlin, Germany (ATLAS.ti 24) for coding to identify recurring phrases and ideas. Direct quotes were incorporated to accurately reflect the participants’ statements. The process began with data familiarisation, followed by defining the unit of analysis and identifying relevant segments.^8^ Inductive coding was used to generate initial themes, which were then organised into categories. Themes were subsequently validated against the data, ensuring rigour through transparency, reflexivity and triangulation. This method allowed for the discovery of meaningful patterns and insights aligned with the research question.^8^

To ensure the quality and trustworthiness of our study, we adhered to established qualitative research criteria, including credibility, transferability, dependability, confirmability and authenticity.^9^ Credibility was maintained through meticulous recording of participants’ perceptions during interviews and the integration of researcher observations to identify influencing factors. Triangulation of information through repetitive questioning further ensured comprehensive data collection. Participants were selected based on their direct experience with CHWs, allowing for a diverse representation of perspectives on CHWs’ roles in health promotion. All data collection instruments, such as field notes and audio recordings, were rigorously documented and reviewed by a supervisor to ensure dependability. We focussed on confirmability by systematically analysing data to faithfully represent participants’ voices. Direct quotes were used to authentically convey participants’ statements, preserving the integrity and transparency of their contributions to the study.

### Ethical considerations

Ethical clearance to conduct this study was obtained from the University of Pretoria, Faculty of Health Sciences Research Ethics Committee (No. 664/2023). The COPC Research Unit and the Melusi community leaders granted permission for the study to be conducted in Melusi community. Individuals who agreed to participate in the study were required to sign an informed consent form.

## Results

The study comprised of 20 participants, with 3 males and 17 females as detailed in Table 1. The ages of the participants, which varied from 28 to 67 years, showed a diversified demographic. Additionally, their duration of residency in Melusi varied from 3 to 22 years, providing a broad spectrum of perspectives within the community.

**TABLE 1:** Characteristics of study participants.

Participant	Age	Sex	Number of years and months residing in Melusi
P1	32	Male	12 years
P2	49	Male	22 years
P3	28	Female	8 years
P4	54	Female	4 years 5 months
P5	39	Female	11 years 1 months
P6	54	Female	7 years
P7	33	Female	3 years 2 months
P8	40	Female	7 years
P9	54	Female	12 years
P10	29	Female	5 years 2 months
P11	29	Female	5 years
P12	29	Female	4 years 6 months
P13	67	Female	5 years
P14	30	Female	8 years
P15	37	Female	7 years
P16	42	Female	8 years
P17	33	Female	7 years
P18	63	Male	7 years
P19	33	Female	14 years
P20	31	Female	5 years

Numerous themes emerged concerning the community’s perception of CHWs and their role in health promotion within Melusi’s informal settlement. These themes include community’s expectations from CHWs, the role of CHWs in addressing health challenges and barriers and recommendations for improvement (Table 2). Each theme offers valuable insights into the dynamics between CHWs and Melusi’s residents, fostering a comprehensive understanding of the community’s viewpoint (Table 2).

**TABLE 2:** Community perceptions and recommendations: Themes and sub-themes from a study on community health workers in Melusi.

Theme	Sub-theme	Summary
Community’s perception about CHWs	Communication Skills and Engagement Strategies	Participants praised CHWs for their effective communication skills and engagement strategies, citing their ability to convey information clearly and engage with community members.
	Overall Positive Experience	Participants expressed overall positive sentiments towards CHWs, highlighting their pleasant interactions and helpful support during health-related challenges.
Community expectations from CHWs	Access to Health Services	Expectations included providing access to health services such as HIV and pregnancy tests, as well as information about nearby clinics and social workers.
	Advocacy and Guidance	Participants expected CHWs to advocate for health issues, provide guidance on maintaining well-being, and assist with educational support for children.
Role of CHWs in addressing health challenges	Guidance and Referrals	The CHWs provided guidance and referrals for community members to start treatment, promoting acceptance of health conditions, and addressing instances of substance abuse.
	Medication Adherence and Support	The CHWs supported medication adherence, ensured access to medication during household visits, and provided comprehensive care beyond medical services.
	Health Assessments and Education	Participants highlighted CHWs’ role in conducting health assessments directly from homes and educating community members about health-related issues.
Barriers faced by CHWs	Resistance and Hostility	The CHWs encountered resistance, hostility, and verbal abuse from community members, hindering their ability to provide effective healthcare services.
	Working Hour Constraints	Challenges included limited working hours, making it difficult for CHWs to reach households where occupants are employed during those hours.
Health challenges faced by the community	Communicable Diseases	Most pressing health challenges included communicable diseases such as HIV, TB, and STIs, which posed significant health risks to the community.
	Non-Communicable Diseases	Other prevalent health challenges included non-communicable diseases such as diabetes and hypertension, along with mental health issues such as depression.
Recommendations for community awareness	Health Education Workshops and Community Meetings	Recommendations included organising health education workshops and community meetings to raise awareness about the role of CHWs and promote community health literacy.
	Establishing Focus Groups	Participants suggested establishing focus groups to educate the community about health-related issues and life skills, fostering a deeper understanding of health and well-being.
	Ensuring Confidentiality	Suggestions included reassuring residents about the confidentiality of discussions with CHWs, fostering trust and encouraging open communication about health concerns.
Suggestions for improving CHW services	Increasing CHW Workforce	Recommendations included hiring more CHWs to meet the growing healthcare needs of the community and enhance the effectiveness of healthcare delivery.
	Extending Clinic Hours and Facilities Expansion	Suggestions included extending clinic hours, hiring more staff, and expanding facilities to improve access to healthcare services and accommodate community needs.

CHWs, Community health workers; HIV, human immunodeficiency virus; TB, tuberculosis; STI, sexually transmitted infection.

### Community’s interaction and perception about community health workers

Participants commended CHWs for their effective communication skills and capacity to deliver invaluable health services:

‘They know how to speak to people and they know how to explain themselves. They are good when it comes to come to getting attention from a person. They service is just good.’ (P20_F_31)

‘The care workers once visited me, they spoke to me in a nice manner and with smiles.’ (P10_F_29)

‘Yes, the interaction was amazing and they were all so informative about the questions I had for them.’ (P12_F_29)

One participant remarked on the CHWs’ courteous demeanour and thoroughness during household visits, while another acknowledged the timely and helpful support provided during a challenging situation involving a sick family member:

‘They are good people, they spoke nicely with us and they counted us to know how many we are in the house.’ (P17_F_33)

‘The visit was right, because I had a sick family member and they assisted me with her.’ (P4_F_54)

Participants admired CHWs’ determination in their door-to-door efforts:

‘They persevere and reach people with their door-to-door initiative even when it is difficult.’ (P5_F_39)

### Community expectation from community health worker

Expectations were voiced regarding CHWs providing access to health services, which included offering information about nearby clinics, conducting HIV and pregnancy tests, screening for TB and diabetes during household visits, and facilitating connections between community members and social workers. When asked about the health-related services or support they expect from CHWs, the participants responded:

‘HIV testing and testing for diabetes and also TB (tuberculosis). Most people around are coughing a lot.’ (P19_F_33)

‘They should help the sick, they should inform us about the clinics near us. They should also give us information on where we can get help from social workers.’ (P11_F_29)

Participants expressed expectations for CHWs to advocate for health issues and provide guidance on maintaining well-being:

‘They must help with sick family members, ensure they eat well, and adhere to their treatment.’ (P3_F_28)

There was an expectation for CHWs to assist with children not attending school, providing educational support and guidance:

‘I expect them to assist with children who are not attending school. Those children need education.’ (P17_F_33)

One participant anticipates collaboration between CHWs and nurses, particularly in instances where the clinic is inaccessible, to provide assistance to the community across various needs:

‘They should do their community rounds with nurses, due to the fact that sometimes the clinic isn’t available. They also assist the community with any other help they might need from them.’ (P14_F_30)

### Role of community health workers in addressing health challenges

In this theme, participants shared their observations of how CHWs address health challenges. Perceptions from participants suggest that CHWs contribute by providing guidance and referrals for community members to start treatment:

‘They also assisted a lot of people to start treatment.’ (P11_F_29)

‘Okay, my mother was unwell and sometimes was not able to go to the clinic, so one care worker assisted with referring her to the clinic, and till today she doesn’t suffer whenever she goes to the clinic.’ (P12_F_29)

‘They came to me and asked for my information and status, and I disclosed. They asked me if I am on treatment or not. When I said I was not on treatment they encouraged me to go to the clinic a check if I am not yet on a stage where I should be starting treatment, likely I got there right on time and I was put on treatment. Ever since I have started everything is going well I am healthy.’ (P6_F_54)

They support in treatment adherence, ensuring that household members who are unable to reach health facilities receive their medication during household visits:

‘They also bring some patients pills when they cannot go collect for themselves.’ (P14_F_30)

Participants highlighted instances where CHWs played a role in promoting acceptance of health conditions, especially in cases related to HIV:

‘There was a sick lady who was sick but did not go to the clinic to seek help and she was not on any treatment. She was coughing, those people came and talked to her and encouraged her to go to the clinic. She is now doing very well, you will not even tell it’s her.’ (P5_F_39)

Participants underscored the comprehensive role of CHWs, including addressing instances of abuse within the community:

‘They helped someone to escape abuse, they referred the person to a social worker so that that person can get the help they needed.’ (P11_F_29)

Participants also underscored the significance of CHWs in conducting health assessments directly from homes:

‘[*W*]hen there is a mobile clinic they pass by me and when they do health checks they also make sure I also get checked.’ (P1_M_32)

‘They help with doing health checks and testing from the homes since most community members are afraid of going to the clinic.’ (P3_F_28)

The CHWs were commended for their holistic approach to community well-being. Beyond medical services, they extend support in obtaining social benefits, such as providing food parcels:

‘They helped us with getting food parcels and vouchers which was a very great initiative and we are grateful.’ (P1_M_32)

‘They assist with food parcels and mostly they have and know relevant solutions and information to any problems we might have in the community.’ (P13_F_67)

One participant recounted an impactful intervention by CHWs, where they provided guidance and support to a vulnerable child in the community:

‘They once helped this other child who did not have a birth certificate, not in school, and abusing alcohol. This child was so wrong that she would swear at her parents always at the tavern drinking and even changing boyfriends. They came and encouraged her that education is the key and tavern will not pay her, they also gave her an advice that having different partners is not good she will end up being sick. They guided her to go to school so she can succeed in life.’ (P10_F_29)

Another participant highlighted CHWs’ educational outreach efforts regarding alcohol abuse and school attendance during household visits:

‘[*T*]hey visit households and educate them on the dangers of alcohol abuse and the consequences of not staying in school.’ (P10_F_29)

### Barriers and recommendations for improvement

#### Challenges faced by community health workers

Participants recounted situations where CHWs encountered resistance, hostility or rudeness from community members, including instances where some individuals refused to open their gates or doors, while others even chased them away:

‘Community members do not want them, they are not welcoming them into their home others even close their gates.’ (P13_F_67)

Participants continued to highlight the difficult circumstances faced by CHWs, observing instances where they were subjected to verbal abuse and insults, and encountered young children in vulnerable situations because of the absence of parental care:

‘Most households are not welcoming and do not treat them well. Some chase them away, others shout at them and swear at them.’ (P11_F_29)

‘People swear at them, they insult them and, in some cases, they find young children with parents who have long passed away, this comes as a barrier because the children are too young to take care of themselves.’ (P16_F_42)

Participants expressed concerns about the confidentiality of their health information when interacting with CHWs, with one noting uncertainty about specific issues but highlighting apprehensions regarding the disclosure of illnesses to others:

‘I do not know what specific problems they are facing. But I think some people don’t like them because some say they disclose their illnesses to other people.’ (P4_F_54)

Another participant emphasised the importance of maintaining confidentiality to enable individuals to fully disclose their health concerns and receive assistance:

‘People cannot disclose to the community health workers and this hinders them to do their job in the community.’ (P6_F_54)

‘According to how I know their work and since we have different sicknesses, I would appreciate it if they can assist those who cannot help themselves and also what is spoken with every patient should be kept confidential at all times.’ (P6_F_54)

Participants stressed the challenges faced by CHWs, who operate within designated working hours, in reaching households where occupants are employed during those hours:

‘In cases where there is a severely sick person in the household, they cannot come and make sure that the patient takes their medication on time since they have working hours (they do not work 24/7.’ (P1_M_32)

‘They should work full-time for elderly people and for chronic especially those living with HIV, people mostly do not accept their statuses and do not take their treatments well.’ (P7_F_33)

### Health challenges faced by community

The majority of participants identified communicable diseases, particularly HIV, TB, and Sexually Transmitted Infections (STIs), as the most pressing health challenges faced by residents of the informal settlements in the City of Tshwane:

‘Mostly its HIV, they are struggling with HIV.’ (P8_F_40)

‘The first and high illness is HIV/AIDS followed by STIs, TB and flu is also common.’ (P20_F_31)

Diabetes and hypertension were the two non-communicable diseases (NCDs) that participants pointed out among the most pressing health challenges faced by residents, followed by depression:

‘High Blood Pressure and sugar diabetes. Is depression a health problem?’ (P7_F_33)

‘We can include depression, most ladies who are staying with their boyfriends suffer from depression. It is a big challenge.’ (P7_F_33)

### Recommendations for community awareness

To enhance the recognition and acceptance of CHWs within the community, a participant recommended organising health education workshops and community meetings to raise awareness about the role of CHWs:

‘If only we can have health education workshops to educate the community about health workers and their roles in the community.’ (P3_F_28)

In addition, it was recommended that CHWs should establish focus groups to educate the community about health-related issues and life skills:

‘They can also create focus groups where they can educate us about health-related issues and life.’ (P10_F_29)

Emphasising confidentiality, a participant proposed a community meeting to reassure residents about the privacy of discussions with CHWs:

‘I think there should be a meeting with the community, to reassure them that all things discussed with community health workers will remain highly confidential.’ (P4_F_54)

One participant recommended the hiring of more CHWs, accentuating their value and the community’s need for their assistance:

‘I would recommend that they hire more community health workers, they are very helpful and we need them.’ (P5_F_39)

Another participant recommended extending clinic hours, hiring more staff, and expanding facilities:

‘I can recommend that our clinic can improve and work 24hrs, also that they should hire enough health workers and if we can have nurses and a doctor and they should extend the clinic.’ (P11_F_2)

## Discussion

This study involved 20 participants to explore community perceptions of CHWs and their role in health promotion. The participants in this study were mainly female (85%). Participants praised CHWs for their communication skills, engagement strategies and ability to provide valuable health services. Community viewed CHWs not just as sources of information but also as supportive figures during health-related challenges. Community members expressed various expectations from CHWs, including healthcare services, educational support, advocacy for health issues and guidance on well-being. Despite these positive perceptions, participants found barriers such as community resistance, lack of privacy and difficulties in reaching households, which hindered CHWs’ effectiveness. However, CHWs were commended for their impact on community well-being, offering health services, emergency support, food assistance and improved healthcare accessibility.

The findings show that study participants from the Melusi informal settlement had positive perceptions of CHWs, highlighting their role as valuable resources in the community. The positive perception findings of this study align with the consistent evidence from previous research, which has continually emphasised the favourable perceptions of CHWs.^10^ Inadequate communication disrupts operational processes and fosters mistrust among various stakeholders involved in patient care.^11^ Community Health Workers are often valued for their ability to communicate effectively with community members and provide culturally appropriate health education and support.^12^ In this study, participants praised CHWs for their communication skills and engagement strategies. This emphasises the importance of effective communication and rapport-building in CHWs’ work, which is consistent with the existing literature.^13^ The results indicate that positive communication, accessibility and a community-centred approach contribute to a favourable perception of CHWs within the community.

The CHWs were not only seen as information sharers but also as supportive figures during health-related challenges in the community. Their role and being trusted in the community have been shown to be important in delivering health promotion and behaviour change.^14^ Participants in the study expressed expectations for CHWs to provide access to health services, advocate for health issues, offer guidance on maintaining well-being and assist with educational support for children. Other studies have also highlighted the varied expectations of community members from CHWs, which include providing healthcare services, advocating for health issues and linking community members to healthcare and social services.^11,14,15,16^

This serves as a reflection of the community’s desire for proactive health promotion. It highlights the urge for community members to be guided regarding healthier lifestyles by CHWs.

The CHWs are often seen as a link connecting communities and formal healthcare systems, addressing gaps in access and service.^15^ This expectation shows that CHWs go beyond providing services, also helping to build communities and connect them to health resources.

The study participants highlighted that the role of CHWs is important, especially in promoting health in the informal settlement of the City of Tshwane. The community sees CHWs as health educators and are of the view that it is important for them to share health information about health issues faced by community members. This aligns with the proactive role of CHWs in health promotion. The findings in this study show that CHWs play an important role in advising on medication adherence, offering advice during household visits and providing essential health services. The CHWs are often involved in a range of activities, including disease prevention, health promotion and social support.^11,14,16^ Their proactive approach, including door-to-door visits and delivering pills, ensures access to medications and healthcare services, contributing significantly to community health.^11,16^

The positive impact of CHWs on community well-being, including their role in providing essential health services, promoting acceptance of health conditions and supporting clinic visits, is well-documented in the literature.^11,17^ The CHWs have been shown to improve health outcomes, increase healthcare access and empower communities to take control of their health.^11,18^ The results of this study show that they provide not only health services but also emergency support, food assistance, enhanced healthcare accessibility and educational initiatives. In line with the other studies, CHWs encourage individuals to seek treatment, promote acceptance of health conditions, and support clinic visits and medication adherence, particularly in cases related to HIV as demonstrated in this study.^11,16^

Despite community members having positive perceptions regarding CHWs, the study also identified several difficulties that prevent CHWs from rendering services. These included community resistance, a lack of privacy and confidentiality, and difficulties in reaching households. This concurs with what other studies have found, which is that CHWs encounter various challenges in their role such as resource constraints, a lack of recognition and inadequate support of the health system.^16,19^ The CHWs play a vital role in improving health outcomes and reducing health differences in communities that don’t have enough healthcare.^20^ The however, often struggle to make community members trust them, understand their cultural beliefs and access resources to effectively carry out their duties.^21,22^ These challenges highlight the need for strategies to support CHWs overcome the difficulties and improving their effectiveness in the community.

The community members’ fear of breach of confidentiality highlights the need to reinforce the importance of confidentiality among CHWs and also raise awareness in the community about the ethical standards CHWs maintain when handling sensitive health information. Working on protecting community confidentiality and addressing challenges in open communication can further enhance the effectiveness of CHWs in the community as suggested by a participant in this study. Expectations for confidentiality emphasise the ethical considerations in CHWs’ interactions. Participants acknowledged CHWs’ engagement beyond healthcare.

This study shows that there are constraints in CHWs’ working hours, which create a barrier to reaching certain community members. Having a different approach such as CHWs working with flexible scheduling might help address this challenge.

Participants in the study from the informal settlements of the City of Tshwane mentioned a wide range of health issues. Communicable diseases such as TB, HIV, and STIs were considered to be major health risks within the community. Health interventions are required to enhance patient outcomes and prevent the disease from spreading.^17^ It is necessary to increase health awareness and prevent these illnesses because of the possible health risks associated with health.^17^ In addition to infectious diseases, NCDs such as diabetes and hypertension were shown to be prevalent health issues. The NCDs are becoming more prevalent because of poor lifestyle choices and limited access to healthcare.^23^

To increase CHWs’ efficacy in promoting health in the City of Tshwane’s informal settlements, study participants offered several suggestions. A crucial suggestion was to arrange community gatherings and workshops on health education to increase understanding of the function of CHWs and advance community health literacy. This recommendation highlights the value of community involvement and education in enhancing health outcomes and giving residents the power to take charge of their health.^15^

Participants recommended that in the community meetings, residents should be reassured on the confidentiality of their conversations held with between community member and CHWs. This recommendation emphasises the value of confidentiality and trust in healthcare interactions because it can motivate residents to ask for assistance and be transparent about their health concerns.^24^

Setting up focus groups to inform the public about health-related concerns and life skills was another suggestion. This recommendation emphasises the value of community-based learning and the part CHWs play in helping community people gain a greater understanding of health and well-being.^25^

## Conclusion

This study emphasises how important CHWs are in promoting health in the district of the City of Tshwane informal settlements particularly Melusi. The positive perception of CHWs by community members highlights how they are valuable resources in the community. The CHWs are essential in addressing health challenges, even though CHWs face challenges such as community resistance, hostility from community members, concerns about confidentiality and difficulties reaching households during non-working hours. To improve the situation, participants recommended having health education workshops, community meetings and focus groups to raise community awareness and enhance community understanding of CHWs’ roles. They also suggested increasing the visibility of CHWs in the community and hiring more CHWs. In general, CHWs play an important role in preserving community access to health, raising health awareness and improving overall community health and well-being.

## Strength and limitations

Achieving data saturation in this study was crucial to comprehensively capture the study topic, suggesting minimal additional insights from further data collection. The selection of participants was a strength, enhancing representativeness and providing diverse perspectives on CHWs in the community. Direct interaction with CHWs and health promotion activities enriched understanding of community views. Detailed descriptions of the study setting and participant selection process bolstered the transferability of findings, offering a thorough account of perceptions in the Melusi informal settlement and facilitating comparisons with other communities’ views on CHWs.

The study encountered logistical constraints in locating participants’ addresses, particularly in Melusi, an informal settlement where some residences lack formal addresses. These challenges hindered the ability to effectively contact and include all intended participants, potentially limiting the study’s scope and generalisability.

The study may not have fully represented the whole viewpoint about informal settlements in the City of Tshwane because the focus was on participant’s perceptions. The study may not have been as applicable to other informal settlements with diverse environment or people because it focussed on a specific informal settlement in the City of Tshwane.

## Implications

Policy implications from the study highlight the need for enhancing CHWs’ communication skills and engagement strategies to effectively convey health information and foster positive interactions with community members. Policymakers should ensure that CHWs meet community expectations by providing essential health services, advocating for health issues and supporting comprehensive care through referrals and educational support. Addressing challenges such as limited working hours and community resistance requires policies that expand capacity and support systems of CHW, aiming to improve health outcomes in managing prevalent health challenges such as communicable diseases, NCDs and mental health issues.
